# DArTseq-based SNP markers reveal high genetic diversity among early generation fall armyworm tolerant maize inbred lines

**DOI:** 10.1371/journal.pone.0294863

**Published:** 2024-04-17

**Authors:** Gloria Boakyewaa Adu, Frederick Justice Awuku, Ana Luisa Garcia-Oliveira, Isaac Kodzo Amegbor, Charles Nelimor, Jerry Nboyine, Benjamin Karikari, Benedicta Atosona, Kulai Amadu Manigben, Paulina Abanpoka Aboyadana

**Affiliations:** 1 CSIR-Savanna Agricultural Research Institute, Nyankpala, Ghana; 2 International Maize and Wheat Improvement Center (CIMMYT), Nairobi, Kenya; 3 Department of Molecular Biology, College of Biotechnology, CCS Haryana Agricultural University, Hisar, Haryana, India; 4 Faculty of Natural and Agricultural Sciences, Department of Plant Breeding, University of the Free State, Bloemfontein, South Africa; 5 Department of Agricultural Biotechnology, Faculty of Agriculture, Food and Consumer Sciences, University for Development Studies, Tamale, Ghana; Nuclear Science and Technology Research Institute, ISLAMIC REPUBLIC OF IRAN

## Abstract

Diversity analysis using molecular markers serves as a powerful tool in unravelling the intricacies of inclusivity within various populations and is an initial step in the assessment of populations and the development of inbred lines for host plant resistance in maize. This study was conducted to assess the genetic diversity and population structure of 242 newly developed S_3_ inbred lines using 3,305 single nucleotide polymorphism (SNP) markers and to also assess the level of homozygosity achieved in each of the inbred lines. A total of 1,184 SNP markers were found highly informative, with a mean polymorphic information content (PIC) of 0.23. Gene diversity was high among the inbred lines, ranging from 0.04 to 0.50, with an average of 0.27. The residual heterozygosity of the 242 S_3_ inbred lines averaged 8.8%, indicating moderately low heterozygosity levels among the inbred lines. Eighty-four percent of the 58,322 pairwise kinship coefficients among the inbred lines were near zero (0.00–0.05), with only 0.3% of them above 0.50. These results revealed that many of the inbred lines were distantly related, but none were redundant, suggesting each inbred line had a unique genetic makeup with great potential to provide novel alleles for maize improvement. The admixture-based structure analysis, principal coordinate analysis, and neighbour-joining clustering were concordant in dividing the 242 inbred lines into three subgroups based on the pedigree and selection history of the inbred lines. These findings could guide the effective use of the newly developed inbred lines and their evaluation in quantitative genetics and molecular studies to identify candidate lines for breeding locally adapted fall armyworm tolerant varieties in Ghana and other countries in West and Central Africa.

## Introduction

Maize (*Zea mays* L.) is a diploid (2*n* = 20) crop with a genome size of 2.365 giga base pair [1]. Among the world’s most important grain cereals, maize ranks third in production following rice and wheat [2, 3]. In Africa, maize is grown on over 40 million hectares of land [4], making it the mainstay of most economies, with consumption ranging between 52–450 g/person/day [5]. However, the existing trends in maize production across Africa are woefully inadequate to meet the ever-growing consumers’ needs [6]. This is because of an array of biotic and abiotic factors that adversely affect grain quality and quantity [7].

The fall armyworm (FAW) [*Spodoptera frugiperda* (J.E. Smith) (*Lepidoptera*: *Noctuidae*)] has recently emerged as an additional threat, attacking maize at all developmental stages [8]. This pest was first detected in central and western Africa in 2016 [9], and since then it has spread throughout sub-Saharan Africa (SSA), wreaking havoc on major staple crops, including maize. Singh et al. [8] opined that the majority of the commercially cultivated maize varieties in SSA are highly susceptible to FAW. Thus, in the absence of any sustainable control measures, FAW could worsen food insecurity and poverty amongst the millions of smallholder farmers who depend on maize cultivation for livelihood [10]. A study by Day et al. [11] of merely twelve of Africa’s nations engaged in maize cultivation revealed that inadequate management of FAW could lead to annual maize production deficits of approximately 8.3–20.6 million tonnes. The total estimated annual cost of FAW to agriculture in Africa may amount to US$ 9.4 billion yearly [12]. In Ghana, maize yield losses from the FAW invasion were estimated to be 28% in 2016, resulting in a total loss of US$ 146 million. National maize yield losses due to FAW increased by up to 40% in 2018, amounting to US$ 177 million [13].

Synthetic pesticides are commonly used to control FAW in Africa due to their immediate effectiveness [14, 15]. However, their use raises concerns about potential harm to humans, the environment, and non-target organisms and the emergence of pesticide resistance, as well as resulting in economic loss due to the high cost for smallholder farmers. The search for sustainable alternatives to control FAW pests [11, 16, 17], such as host plant resistance (HPR) through breeding, is economically viable, readily available, and environmentally conscious [18]. Fall armyworm tolerant maize varieties can produce good yields even when attacked by the pest. They use HPR, a natural way of resisting FAW damage that is compatible with other control methods and aligns seamlessly with the principles of integrated pest management (IPM) [19].

In this regard, crop diversity has significant implications for how plants interact with pests and their ability to defend against pest attacks. Therefore, having adequate knowledge of the extent of genetic diversity within and among breeding populations is crucial for the success of any genetic improvement programme. The extensive diversity inherent in maize requires an adept system for capturing the suitable germplasm required to establish a population or breeding pool. DNA-based molecular markers, among the genomic tools, offer an efficient approach to harnessing diverse variations from various sources. These tools facilitate the introgression of valuable traits into breeding pipelines [20]. In maize, molecular markers have been widely applied for diversity analysis to categorize inbred lines into heterotic groups, select diverse parental combinations to generate segregating progenies, identify efficient testers for evaluating inbred lines in hybrid combinations, identify desirable genes from diverse germplasm sources and monitor line purity and genetic identity [21–23].

Earlier studies on genetic diversity analysis in maize were mostly based on either phenotypic or low-throughput molecular markers such as amplified fragment length polymorphisms (AFLPs) [24], randomly amplified polymorphic DNA markers (RAPDs) [25], restriction fragment length polymorphisms (RFLPs) [26] and simple sequence repeats (SSRs) or microsatellites [22, 27–30]. With advances in high-throughput genotyping platforms such as genotyping by sequencing (GBS), single nucleotide polymorphisms (SNP) are presently the marker of choice for diversity analysis due to their bi-allelic nature and ability to be expressed at a much higher frequency in the genome than SSRs and other markers. Likewise, genotyping of SNPs can easily be automated [31]. Single nucleotide polymorphism markers have been used in maize and other cereal crop breeding programmes for various purposes. Previous research employing SNP markers has demonstrated the potential of SNP markers as an efficient tool for examining the genetic diversity, population structure, and purity of different types of maize varieties, such as inbred lines and FAW resistance varieties [32–35]. Additionally, SNP markers can be used to elucidate and understand the population dynamics of FAW strains in Africa [36–39].

The aim of this study was to evaluate the genetic diversity and population structure of 242 newly developed S_3_ inbred lines, utilizing SNP markers. Additionally, the study sought to determine the level of homozygosity attained within each of the 242 inbred lines. The findings from this study will play a pivotal role in selecting parental lines and will also serve as a foundation for further quantitative genetics and molecular analyses. These endeavours are geared towards the development of FAW tolerant varieties tailored for local conditions in Ghana and other West and Central African (WCA) nations.

## Materials and methods

### Planting materials

The 242 S_3_ inbred lines used in this study were developed at the Nyankpala Experimental Station by CSIR-SARI (S1 Table). The development of the inbred lines began in 2019 with 187 FAW tolerant source populations obtained from the International Corn Foundation (ICF) and the Korea Food for Hunger International Center in Zimbabwe (S1 Table). The base germplasm from which the 187 source populations of the inbred lines were generated was developed by the International Institute of Tropical Agriculture (IITA) for lowland and mid-altitude environments (referred to as IITA base germplasm) and by the International Maize and Wheat Improvement Center (CIMMYT) for mid-altitude environments (referred to as CIMMYT base germplasm). First, the IITA and CIMMYT base germplasms were crossed with five commercial hybrids and open-pollinated varieties (OPVs) from Zimbabwe. Later, Asian downy mildew tolerance materials developed by the ICF maize programme, as well as IITA/ICF grey leaf spot tolerance materials developed for South Asian countries, were incorporated into the populations. Overall, the populations were bred to withstand drought, FAW, *Striga*, maize viruses (maize streak virus and necrotic lethal virus, among others), leaf diseases (blights, rusts, grey leaf spots), and low soil nitrogen conditions. It took nine years to develop the source populations, starting in 2010. The procedures used for the inbred line extraction from the source populations and the generational advancement of the inbred lines were described by Badu-Apraku and Fakorede [40] and the rapid inbreeding techniques described by Chase and Nanda [41].

### DNA extraction and genotyping

Fresh leaves from two-week-old plants were collected by sampling four 6 mm leaf discs from each plant. In total, samples were collected from 242 maize inbred lines. The samples were dried using silica gel, and sent to Diversity Arrays (DA), Australia, for genotyping against the Maize DArTag 3.3K EiB (2.0) panel from the CGIAR CIMMYT-Excellence in Breeding platform (https://excellenceinbreeding.org/toolbox/services/mid-density-genotyping-service). The publicly available panel has a total of 3,305 DarTag markers and encompasses data developed from more than 10,000 maize inbred lines with diverse origins during the last 20 years. The extraction, quality testing, and SNP calling of the samples followed the protocol outlined in Adu et al. [32].

### Data cleaning and structure analysis

Data were cleaned by removing SNP markers with more than 20% missing data, 10% heterozygosity, and a minor allele frequency of less than 2%. This resulted in a total of 1,184 informative SNP markers, which was 34.8% of the SNP markers used for the genotyping of the inbred lines for further analysis. Information on the genetic parameters of the inbred lines and markers was generated using the PowerMarker software v 3.2.5 [42]. These included major allele frequency, gene diversity, heterozygosity, and polymorphic information content.

Structure analysis was carried out with the data from the 1,184 SNP markers using the admixture model with correlated allele frequencies in the STRUCTURE software 2.3.4 [43]. This was done at an initial setting of Markov Chain Monte Carlo (MCMC) of 200,000 and a burn-in period of 100,000 for an assumed subpopulation (K) range of 1 to 10. Each K was run at an iteration of five. To estimate, the maximum delta K (ΔK) for the population, the Evanno method was employed in STRUCTURE HARVESTER [44], an online-based software. A final run for the best K was done at an MCMC of 400,000 and a burn-in period of 200,000 with a single repeat. Inbred lines were assigned to subpopulations at a minimum probability equal to or greater than 60%, while those less than this threshold were assigned as admixtures. Three membership probability levels of 80%, 70%, and 60% were individually evaluated to access the optimum groupings of the test inbred lines. Analysis of molecular variance (AMOVA) was carried out using a Microsoft Excel add-in software GenAlEx 6.503 [45].

### Cluster analyses

The delta K estimated from the STRUCTURE analysis was used to assign the inbred lines into subpopulations and admixtures. Principal coordinate analysis (PCoA) and neighbor joining cluster analysis were carried out in DARWIN software version 6.0.021 [46, 47] after a simple matching dissimilarity matrix was generated at a bootstrap value of 10,000 in TASSEL [48]. Relative kinship analysis was done using TASSEL [48] based on the “pairwise IBS” method.

## Results

**Fig 1:**
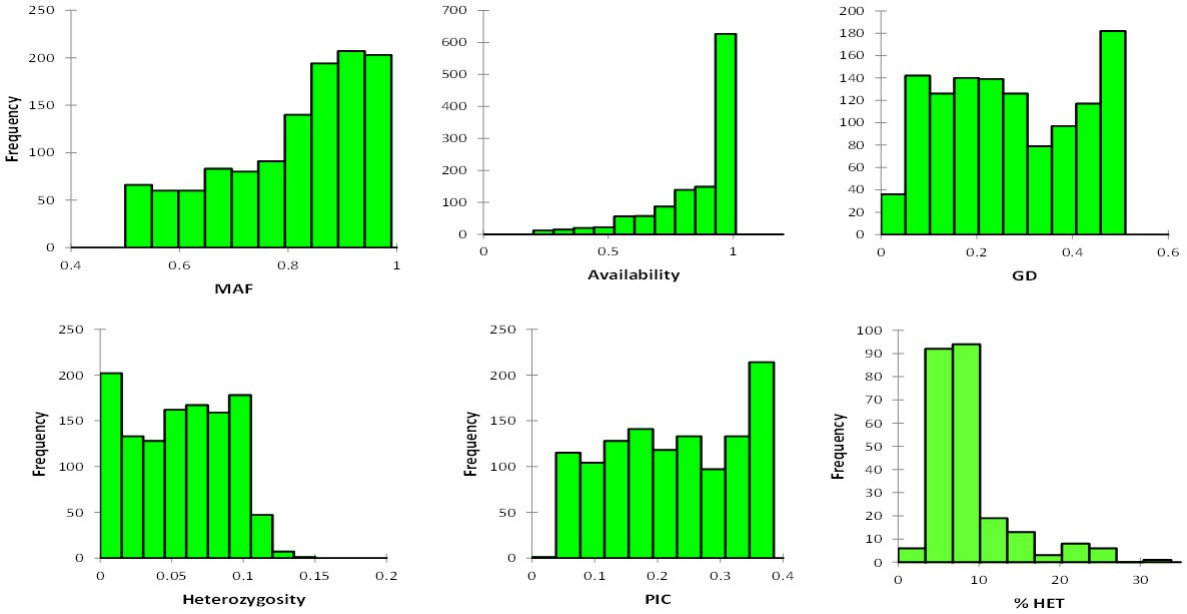
Distribution of informative single nucleotide polymorphism (SNP) marker parameters on the 1,184 SNP markers and heterozygosity of the 242 inbred lines. Gene diversity (GD), Major allele frequency (MAF), Polymorphic information content (PIC), and Percent residual heterozygosity (%HET).

The 1,184 SNP markers obtained after the data cleaning had an availability range of 20–100% and a mean of 86% (Fig 1 and S2 Table). Gene diversity of the markers was high with a maximum of 0.50 and a minimum of 0.04 with a mean of 0.27 (Fig 1). The markers showed a heterozygosity value ranging from 0.00 to 0.14 with a mean of 0.06. The polymorphic information content (PIC) recorded in this study had a minimum of 0.04 and a maximum of 0.38 with a mean of 0.23. Similarly, the major allele frequency ranged from 0.50 to 0.98 with a mean of 0.81. Based on all the 3,305 SNP markers, the residual heterozygosity of the 242 inbred lines ranged from 2.7 to 32.8%, with a mean of 8.8% (Fig 1 and S1 Table).

### Population structure analysis

**Fig 2:**
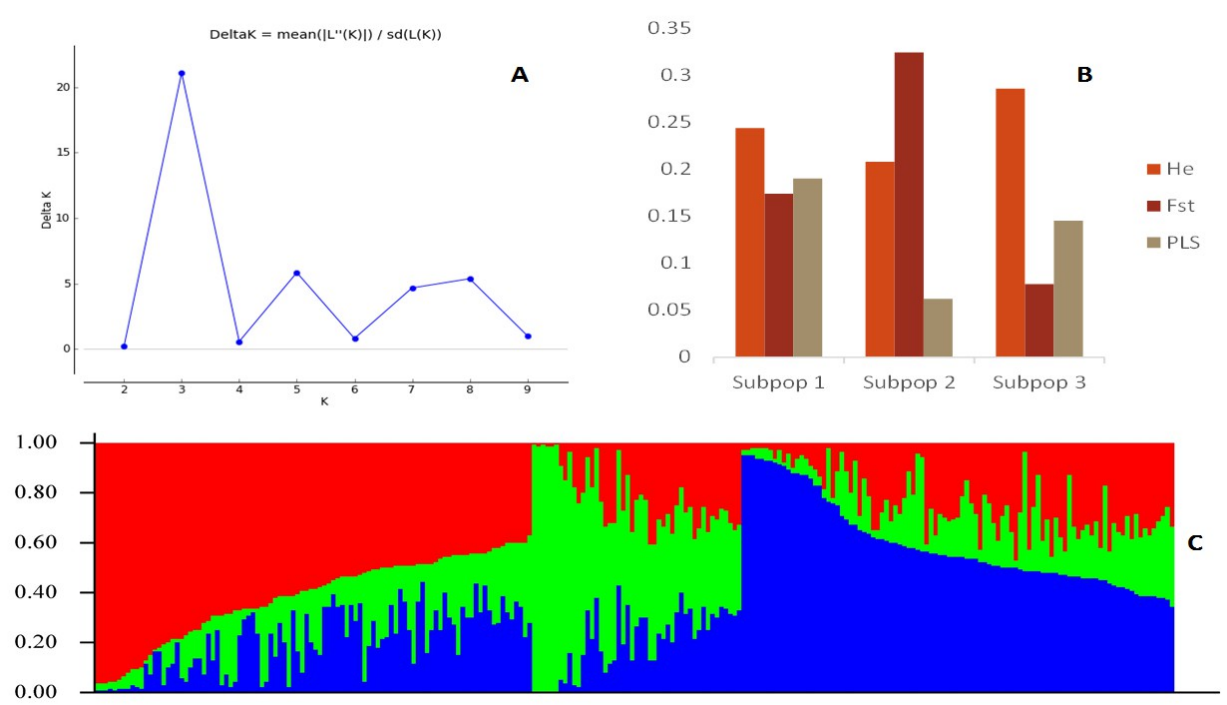
Structure analysis of the 242 inbred lines as revealed by Evanno’s admixture-based method. A: Best delta K for the population structure obtained by the Evanno method was employed in STRUCTURE HARVESTER (Earl and vonHoldt, 2012). B: Population parameters for the subpopulations. Expected heterozygosity (He), Fixation index (Fst), Proportion of inbred lines in each subpopulation (PLS). C: Estimate of structure among the inbred lines with red, green, and blue representing subpopulations 1, 2, and 3, respectively.

The structure analysis of the 242 inbred lines by the admixture model revealed three subpopulations (ΔK = 3) (Fig 2A–2C). At the 80, 70, and 60% probability of association thresholds, 199, 182, and 146 out of the 242 inbred lines were classified as admixtures, respectively (S1 Table). Thus, at the 60% threshold, there were relatively few inbred lines that were unassigned to a specific subpopulation. Using the 60% threshold, 60.3% of the tested inbred lines were admixtures, 19% of the inbred lines (46) were grouped into subpopulation 1, 6.2% of them (15 inbred lines) were grouped into subpopulation 2, and 14.5% of the inbred lines (35) were grouped into subpopulation 3 (S1 Table). Net nucleotide distance, which is a measure of divergence between the subpopulations ranged from 0.03 between subpopulations 1 and 3 to 0.04 between subpopulations 3 and 2. Subpopulations 1 and 2 were the most diverged with a net nucleotide distance of 0.05. Subpopulation 2 recorded the highest fixation index (Fst) (0.32) resulting in its lowest expected heterozygosity of 0.21 (Fig 2B). Subpopulation 3 was the most diverse with a (Fst) of 0.08 and an expected heterozygosity of 0.29 (Fig 2B). Subpopulation 1 falls between subpopulations 2 and 3 with Fst of 0.17 and expected heterozygosity of 0.24.

### Analysis of molecular variance

The analysis of molecular variance (AMOVA) revealed subpopulation differentiation among the inbred lines. The grouping of the inbred lines was per the subpopulations and the admixture groups from the admixture model results. It was observed that there was a higher variation within the subpopulations (97%), while a 3% variation was found among the three subpopulations (Table 1). Pairwise Phi statistic (PhiPT) values among the three subpopulations ranged from 0.04 between subpopulations 1 and 3 to 0.77 between subpopulations 2 and 1. Subpopulations 3 and 2 had a PhiPT value of 0.74. The admixture group shares a distance of 0.15, 0.05, and 0.17, respectively with subpopulations 1, 2, and 3.

**Table 1:** Analysis of molecular variance among 242 maize inbred lines based on 1,184 SNP markers.

Source of variation	DF	SS	MS	Est. Var.	%TV	*p*-value
**Among subpopulations**	3	5612.493	1870.83	22.62	3%	<0.001
**Within subpopulations**	238	195544.81	821.62	821.62	97%	<0.001
**Total**	241	201157.31		844.23	100%	

DF: degree of freedom, SS: sum of squares, MS: mean squares, Est. var.: an estimate of variance, %TV: percentage of the total variation, *p*-value is based on 1000 permutations.

### Cluster analyses

**Fig 3:**
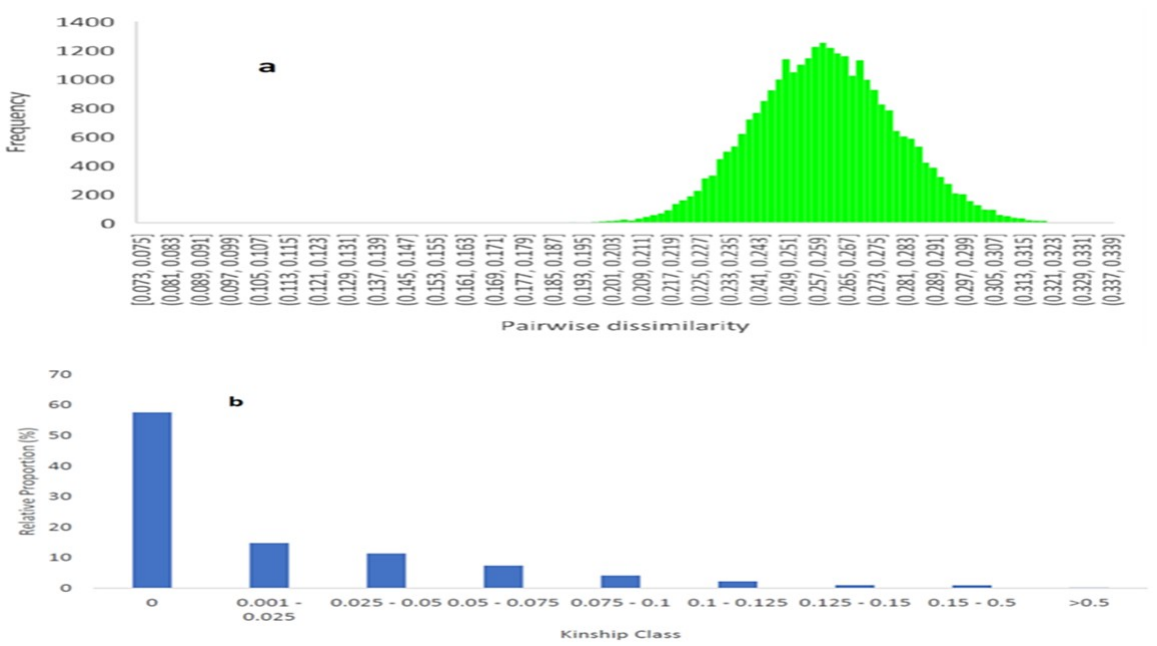
Distribution of pairwise dissimilarity (A) and pairwise relative kinship (B) among the 242 inbred lines based on 1,184 SNP markers.

Both the principal coordinate analysis (PCoA) and cluster analysis were performed using the simple matching dissimilarity matrix. Pairwise dissimilarity among the 242 inbred lines ranged from 0.13 to 0.34 with a mean of 0.22 (Fig 3A and S3 Table). The relative kinship coefficients between pairs of the inbred lines ranged from 0.00 to 1.34 (Fig 3B and S4 Table), with a mean of 0.02. Eighty-four percent of the 58,322 pairwise kinship values obtained in this study ranged from 0.00 and 0.05.

**Fig 4:**
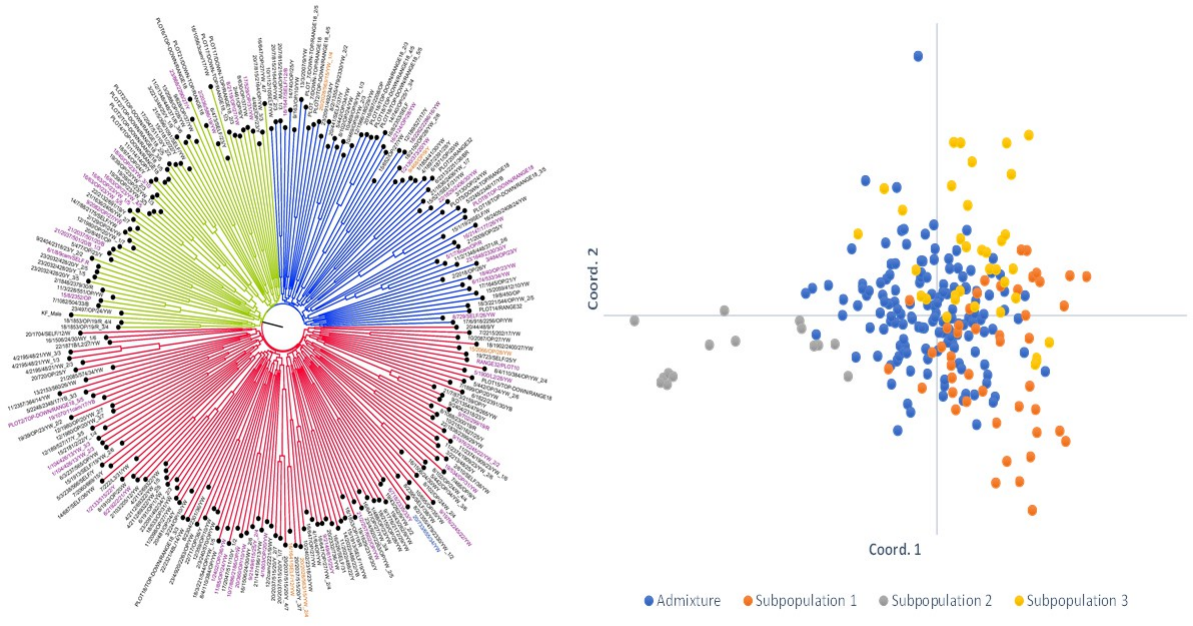
Principal coordinate (Right) and Neighbour Joining clustering (Left) view of the structure of the 242 inbred lines revealed by the 1,184 SNP markers. Colouring of the PCoA and the NJ clustering is based on the grouping of the structure analysis; green represents subpopulation 2, red represents subpopulation 1, and black the admixture group. Names of inbred lines phenotypically classified as highly resistant, moderately resistant, and resistant to FAW are written in black font colour; those classified as tolerant to FAW are written in violet font colour; and those classified as susceptible to FAW are written in brown font colour.

The PCoA and cluster analysis provided a different view of the structure within the population. The PCoA showed that most of the inbred lines were clustered together with a few scattered around (Fig 4). There was concordance in the grouping of the inbred lines as revealed by the structure analysis and the PCoA in terms of the number of groups identified by both methods (S1 Table and Fig 4). The NJ cluster analysis revealed three clusters of the inbred lines, just as identified by the structure analysis. Cluster 1 was made of 13 inbred lines; Cluster 2 consisted of 67 inbred lines and the remaining 167 were grouped into Cluster 3 (S1 Table).

## Discussion

In this study, 242 S_3_ maize inbred lines were assessed for their genetic diversity using 1,184 informative SNP markers: 59% of the markers recorded very high availability (0.90–1.00) across the 242 inbred lines. This suggested that the markers were present in either of two forms (homozygous or heterozygous) for 90% to 100% of the inbred lines. The very high availability recorded showed that the selection of polymorphic markers could be very useful among the inbred lines. The mean PIC of 0.23 recorded in this study indicated that the markers used showed high polymorphism among the inbred lines and could be applied in further selection, and this was almost the same as the PIC of 0.3 recorded in maize by Josia et al. [33]. In contrast, the present study recorded higher PIC than reported in other previous studies [28, 32, 49, 50].

Gene diversity assessed through molecular analysis indicates the extent of genetic diversity present within an examined group of individuals. High gene diversity presents an opportunity to select for wide adaptations to varied constraints and traits. The mean 0.27 gene diversity detected in this study suggested that the diversity among the inbred lines used is moderately high. About 44% of the gene diversity values identified in this study, ranged from 0.3 to 0.50, implying abundant genetic variability within the 242 inbred lines to allow for significant progress in parental line selection, which will result in high genetic gain and heterosis for desired traits. The mean gene diversity (GD) found in this study was comparable with that found by Lu et al. [51] (GD = 0.27), higher than that reported by Adu et al. [32] (GD = 0.22), and Wu et al. [52] (GD = 0.21), but lower than the GD found by Ayesiga et al. [53] (GD = 0.39), and Josia et al. [33] (GD = 0.45). Differences in the set of germplasm used and the number of SNP markers used in the current study and the previous studies cited above may account for the differences in the PIC and GD values [54, 55].

The homogeneity among the tested inbred lines was generally low, with a mean heterozygosity of 8.8%. Only 14% of the 242 inbred lines had heterozygosity levels less than or equal to 5%, 65% of the inbred lines had heterozygosity levels ranging from 5.1 to 10%, and 21% of them had heterozygosity ranging from 10.1% and 32.8%. According to Semagn et al. [22], inbred lines are considered pure or fixed when the proportion of heterozygous SNP loci does not exceed 5%. The higher level of heterozygosity observed for most of the inbred lines used in this study was because they were in their early generation (S_3_) of inbreeding. This finding supports the premise that S_4_ or later-generation maize inbred lines are fixed and pure [33, 51]. However, with 79% of the tested inbred lines having residual heterozygosity of 2.7% to 10%, it is worth noting that the level of homozygosity attained in these inbred lines is relatively higher than what is conventionally expected for S_3_ inbred lines (12.5% heterozygosity) [56, 57]. The rapid inbreeding techniques [41] employed during the generational advancement of the tested inbred lines could have influenced this result. As demonstrated by Chase and Nanda [41], the number of inbreeding generations required to produce homozygotes may be reduced if more homozygous individuals are selected phenotypically in segregating progenies for further inbreeding and also when negative selection for heterotic traits like plant height, days to anthesis, and leaf numbers is practiced.

The structure of a population reveals the inherent groupings within the population [58]. At the molecular level, this presents a stable and reliable grouping of individuals within the panel of genotypes tested, which provides a good selection of inbred lines from different groups for high heterotic hybridizations. The admixture model-based population structure analysis grouped the 242 inbred lines into three subpopulations. Subpopulation 3, which is the least differentiated (Fst = 0.08) of the three subpopulations, conforms to its high heterozygosity (0.29) making it more heterogenous. Therefore, the inbred lines in subpopulation 3 were more diverse when compared to those in subpopulations 1 and 2. The NJ cluster analysis also grouped the inbred lines into three clusters. The PCoA showed similar clustering to the NJ clustering and showed clear separation among the subpopulations as revealed by the admixture model. Thus, the three structure analysis methods provided similar groupings for the 242 inbred lines. The grouping of the inbred lines was mainly based on the pedigree and selection history of the tested lines. The large number of admixtures detected (60.3%) among the inbred lines could be due to, the mixed genetic background and the generation (S_3_ lines) of the inbred lines. At S_3_, there may not have been sufficient generation advancement and selection in those inbred lines to allow the division of the inbred lines into distinct groups. Therefore, additional generations of inbreeding and selection according to a distinct criterion may be necessary before they are well differentiated and could easily be classified into distinct groups. Subpopulation 2 is the most homogeneous of the three subpopulations identified by the admixture model because, despite being the smallest group (15 inbred lines), it had nearly all the inbred lines in the group (87%) originating from the same base germplasm. The inbred lines were extracted from the source populations SP/7/88/19, SP/63/OP/19, SP/40/OP/19, SP/974/OP/19, SP/39/OP/19, SP/129/OP/19, SP/1636/2406/19, SP/2132/691/19, and SP/718/2335/19. All nine source populations originated from the IITA base germplasm. On the other hand, subpopulation 3 had a good representation of inbred lines originating from the IITA (54% of the inbred lines) and the CIMMYT (46% of the inbred lines) base germplasms. In subpopulation 3, the 19 inbred lines derived from the IITA base germplasm included SARI/22/84, SARI/22/117, SARI/22/7, SARI/22/86, SARI/22/36, SARI/22/88, SARI/22/90, SARI/22/154, SARI/22/95, SARI/22/181, and SARI/22/182. Similarly, the inbred lines in subpopulation 3 that were extracted from the CIMMYT base germplasm included SARI/22/83, SARI/22/227, SARI/22/17, SARI/22/221, SARI/22/5 and SARI/22/6. In general, inbred lines with the same parents but different selection histories were grouped into the same cluster by both the admixture and the NJ clustering methods: SARI/22/156, SARI/22/157, SARI/22/158, SARI/22/213, SARI/22/214, SARI/22/215, SARI/22/143, SARI/22/144, SARI/22/145, SARI/22/73, SARI/22/101, SARI/22/212, SARI/22/182 and SARI/22/23. The composition of the three groupings of the 242 inbred lines by the NJ clustering method followed a similar pattern of characteristics as observed in the three subpopulations grouped by the admixture model with regard to the base germplasm they were extracted from. The classification of inbred lines based on the patterns identified in this study is well-known in genetic diversity studies. For instance, Wen et al. [59] used 1,260 SNP markers to classify a panel of 359 multiple stress-resistant maize inbred lines from IITA and CIMMYT into nine subgroups. They discovered that the assignment of the inbred lines to the nine subgroups was based on their pedigree information, environmental adaptations, and breeding schemes. Badu-Apraku et al. [60] classified 439 maize inbred lines developed by IITA and CIMMYT into four groups based on the inbred line’s ancestry, selection history, and kernel colour using 9,642 DArT SNP markers. Semagn et al. [22] used 1,065 SNP markers to classify 450 diverse maize inbred lines developed and used by CIMMYT’s breeding programmes in both Kenya and Zimbabwe into three main subpopulations based on the pedigree of the lines.

Genetic distance quantifies the degree of relatedness among individuals in a population [61], and knowledge of the relatedness of parental lines is crucial in the selection of diverse parents for the highest heterosis in hybrid breeding. The highest pairwise genetic distance among the 242 inbred lines tested in this study was 0.34 (SARI/22/90 and SARI/22/218) and the lowest was 0.13 (SARI/22/204 and SARI/22/71). Most pairwise genetic distances (58%) among the inbred lines were between 0.20 and 0.29, with only 6% of them ranging from 0.30 to 0.34. These results suggested that most of the inbred lines were moderately related, but a good number of them were distantly related. Most importantly, none of them were sister lines, as all 29,164 pairs of the inbred lines had genetic distances greater than 0.05. This finding highlights the fact that, although sharing common ancestors, each of the tested inbred lines has a distinct genetic composition and the potential to contribute new alleles to hybrid development and population improvement programmes. The magnitude of genetic distances obtained in this study is consistent with what was obtained in previous genetic diversity studies by Semagn et al. [22], Ertiro et al. [62], and Dao et al. [63], but lower than what was reported by Josia et al. [33], Adu et al. [32], Semagn et al. [22], and Kondwakwenda et al. [64]. On average, the pairwise genetic distance across inbred lines within subpopulations 1, 2, and 3 identified by the admixture model was 0.18, 0.14, and 0.27, respectively. This finding suggested that inbred lines within the same subgroup seem to have a common ancestor and are genetically related, and vice versa. This further corroborates previous reports that inbred lines in distinct heterotic groups have greater genetic distances between them, and vice versa [60, 65, 66]. Thus, there is a possibility that the three subgroups of the tested inbred lines as identified in this study are the heterotic groupings of the 242 inbred lines. Since all 242 inbred lines are new and have not been extensively field tested in terms of their genetic combining ability, the three subgroups will be maintained as the interim heterotic groups of the inbred lines until discrete heterotic groups are established. The genetic distances between SARI/22/218 and 84 other inbred lines including those listed below were among the pairs with the highest pairwise dissimilarity of 0.30 to 0.34: SARI/22/227, SARI/22/233, SARI/22/238, SARI/22/42, SARI/22/2, SARI/22/237, SARI/22/235, SARI/22/210, SARI/22/155, SARI/22/234 and SARI/22/112. These eleven inbred pairs are among the most genetically divergent combinations that could be exploited for hybrid development and population improvement for multiple stress tolerance including FAW tolerance.

The relative kinship coefficient reflects the approximate degree of identity between two given individuals. Values close to zero indicate a lack of relationship, while those greater than or equal to 1 indicate a complete relationship. Kinship analysis of the inbred lines tested in this study showed consistency with Nei’s genetic distances generated in Darwin software. The pattern of similarities among the inbred lines revealed by both methods was similar. A total of 84% of the 58,322 pairwise kinship coefficients among the inbred lines were near-zero (0.00–0.05), with 58% equal to 0.00 and 26% ranging from 0.001 to 0.05. Only 0.3% of the pairwise kinship coefficients fell above 0.5. These results suggested that most of the tested inbred lines were unrelated or distantly related, indicating no redundant inbred lines. This low relatedness among the tested inbred lines was expected given their diverse genetic backgrounds, which include tolerance to drought, FAW, *Striga*, maize viruses, leaf diseases, and low soil nitrogen conditions. The fraction of near-zero pairwise kinship values found in the current study was substantially higher than that reported by previous researchers. Hao et al. [67] reported that 66.6% of pairs of 80 drought-tolerant maize inbred lines had near-zero kinship coefficients among them. Dao et al. [63] found near-zero kinship values for 61% of pairs of 100 temperate and tropical inbred lines from the Institute of Environment and Agricultural Research (INERA), CIMMYT, and IITA. Also, de Faria et al. [68] reported near-zero kinship values for 10.80% of pairs of 182 tropical maize inbred lines from the public breeding programme of the Universidade Federal de Viçosa in Brazil. However, the proportion of near-zero kinship coefficients obtained in this study is lower than that reported by Wu et al. [69]. The authors found that around 62% of pairwise kinship coefficients among 544 CIMMYT inbred lines were equal to zero, and 33% of them ranged between 0 and 0.05. There is a very low level of relatedness among the inbred lines studied by Wu et al. [69] as compared to those used in this study because the inbred lines used by Wu et al. [69] were more diverse than those used in this study. The inbred lines they used represented a global maize collection of highly diverse inbred lines that were derived from broad-based populations from diverse origins and adapted to different environments across the world.

Analysis of molecular variance revealed higher variability at the molecular level within the grouping (97%) of the admixture model than among groups (3%). This result is peculiar to outcrosses, which show a higher degree of variability within populations than among populations [53, 70]. The result is consistent with the findings of Nelimor et al. [71], Mathiang et al. [72], Ayesiga et al. [53], and Zawadi et al. [73] who reported 83–97% variability within maize populations. The presence of significantly higher variation within the three subpopulations of the tested inbred lines enables the effective selection of core sets of inbred lines that capture the maximum allelic richness of the respective subpopulations, resulting in the identification of genotypes with desirable traits [74].

## Conclusion

Breeding for a desirable trait in maize requires selecting donor parents that do not only possess wide genetic variability but are also distantly related. In this study, moderately high genetic diversity was noticed among 242 S_3_ inbred lines with varying levels of FAW tolerance using 1,184 SNP informative markers. Although the majority of the breeding lines were moderately related, none of them were redundant. The 58,322 kinship coefficients obtained among pairs of the 242 inbred lines were very low, with only 0.3% falling above 0.5%. This indicated the distinctive nature of the vast majority of the inbred lines and their ability to contribute novel alleles to a breeding programme when used. At this stage, these unique lines could be utilized for variety development and population improvement aimed at multiple-stress tolerance such as FAW, drought, and low soil nitrogen tolerance. The 242 inbred lines were grouped into three sub-populations by both the admixture-based model and the NJ clustering methods. The grouping of the inbred lines was based on their pedigree and selection history. The breeding lines are new and do not have established heterotic groupings. So, the three subgroups identified among the inbred lines could be considered the interim heterotic groupings of the inbred lines. Furthermore, the combined use of information on the pedigree as well as the just-established sub-grouping of the inbred lines could guide the selection of genetically divergent parents for hybrid development and the prediction of hybrid performance. In that regard, the eleven inbred pairs listed below were among the most genetically divergent inbred pairs that would be best for heterotic crosses and population improvement purposes: SARI/22/227 and SARI/22/218, SARI/22/233 and SARI/22/218, SARI/22/238 and SARI/22/218, SARI/22/42 and SARI/22/218, SARI/22/218 and SARI/22/2, SARI/22/237 and SARI/22/218, SARI/22/235 and SARI/22/218, SARI/22/218 and SARI/22/210, SARI/22/218 and SARI/22/155, SARI/22/234 and SARI/22/218, and SARI/22/218 and SARI/22/22/112. Conversely, parental combinations between SARI/22/219 and about 39 of the other inbred lines that recorded the lowest pairwise genetic distances (0.13 to 0.19) (S3 Table) must be avoided in hybrid development.

Generally, the 242 inbred lines used in this study were less homogeneous, with only 14% having heterozygosity levels of ≤ 5%. To obtain fixed inbred lines for maximum heterotic effects in conventional hybrid development, additional generations of inbreeding of the lines are needed. However, some of the 208 inbred lines identified to possess high heterozygosity levels could be maintained as early generation lines for pre-breeding and population improvement programmes, as well as for the development of non-conventional hybrids and other kinds of varieties. The 34 inbred lines identified to be fixed could be selected for early generation testing in hybrid combinations, while the other inbred lines undergo additional cycles of inbreeding. This approach would allow breeders to use breeding resources judiciously and identify superior parents for developing productive hybrids faster, compared to if breeders had no information on the level of homozygosity attained in each inbred line and had to take all the tested inbred lines through three additional cycles of inbreeding (from S_3_ to S_6_) before testing them in hybrid combinations.

Ultimately, the information obtained on the inherent genetic diversity and population structure within the 242 inbred lines based on the 1,184 informative SNP markers would aid in the conduct of quantitative genetics and molecular studies, including field testing of the inbred lines in combining ability studies to establish discrete heterotic groupings of the inbred lines and also identify suitable parental line combinations for variety development.

## Supporting information

S1 Table Summary of the pedigree and the percentage heterozygosity levels of each of the 242 inbred lines and the population structure grouping of the inbred lines revealed by 1,184 SNP markers. (XLSX)

S2 Table Summary statistics of the 1,184 informative SNP markers used in the present study. (XLSX)

S3 Table Pairwise dissimilarities among the 242 maize inbred lines used in the present study. (XLSX)

S4 Table Pairwise relative kinship coefficients among the 242 maize inbred lines used in the present study. (XLSX)
